# Novel dual-function GC/MS aided ultrasound-assisted hydrodistillation for the valorization of *Citrus sinensis* by-products: phytochemical analysis and anti-bacterial activities

**DOI:** 10.1038/s41598-023-38130-9

**Published:** 2023-08-02

**Authors:** Roudaina Abdel Samad, Nada El Darra, Alissar Al Khatib, Hadi Abou Chacra, Adla Jammoul, Karim Raafat

**Affiliations:** 1 Faculty of Health Sciences, Beirut Arab University, Tarik El Jedidah, Riad El Solh, P.O. Box: 115020, Beirut, 1107 2809 Lebanon; 2 Department of Industrial Engineering and Engineering Management, Faculty of Engineering, Beirut Arab University, Riad El Solh, P.O. Box 11-5020, Beirut, Lebanon; 3 Food Department, Lebanese Agricultural Research Institute, P.O. Box 2611, Fanar, Beirut, 1107 2809 Lebanon; 4 Phytopharmacy Laboratory, Ministry of Agriculture of Lebanon, Kfarchima, Lebanon; 5 Department of Pharmacognosy and Natural Products, Faculty of Pharmacy, Pharos University in Alexandria, Alexandria, Egypt

**Keywords:** Secondary metabolism, Environmental economics, Chemical biology, Drug discovery, Microbiology, Chemistry

## Abstract

A huge-amount of citrus by-products is being wasted every-year. There is a high-need to utilize these by-products with high-efficiency. This study focuses on the essential oil (EO) isolation from the zest of *Citrus sinensis* (CS) by-products, using a novel dual-function gas-chromatography mass-spectrometry optimized ultrasound-assisted hydrodistillation-prototype (DF-GC/MS-HUS). The CS-EO was GC-analyzed by MS-detector (GC/MS) and optimized by flame-ionization detector (GC/FID). Ultrasound-assisted hydrodistillation (HUS) had a dual-function in CS-EO isolation by utilizing an adequate-energy to break-open the oil-containing glands, and by functioning-as a dispersing-agent to emulsify the organic-phase. The most effective DF-GC/MS-HUS optimized-conditions were isolation under 38 °C and 10 min of 28.9 Hz sonication. The main-components of CS-EO were limonene, β-myrcene, and α-pinene (81.32%, 7.55%, and 4.20%) in prototype, compared to (60.23%, 5.33%, and 2.10%) in the conventional-method, respectively. The prototype CS-EO showed natural antibacterial-potentials, and inhibited the bio-film formation by *Staphylococcus aureus*, *Listeria monocytogenes*, and *E. coli* more-potent than the conventional-method. Compared to conventional-method, the prototype-method decreased the isolation-time by 83.3%, lowered energy-consumption, without carbon-dioxide production, by reducing isolation-temperatures by more-than half, which protected the thermolabile-components, and increased the quantity by 2514-folds, and improved the quality of CE-EO composition and its antibacterial-potentials. Therefore, the DF-GC/MS-HUS prototype method is considered a novel green-technique that minimized the energy-utilization with higher-efficiency.

## Introduction

*Citrus sinensis* (CS) fruit production has been considered one of the first natural products in the eastern Mediterranean region. This fruit grow on the southern half of the Lebanese coast and represent half of the total agricultural output in the country. CS production increased over the year 2001 and peaked in 2004 to reach a production of 395,300 tons. Due to the war in 2006, this production decreased to stand to range of 230,497 tons in 2013^1^. In manufacturing, these fruits are utilized for juices (fresh or commercial) or citrus-based production, in consequence, a huge amount of waste, mostly peels, are trashed. Industries seek a waste management procedure to minimize the costly disposal of these waste materials^2^.

Food waste has always been a worldwide problem, where a huge number of by-products annually get lost and unused. Isolating essential oil from citrus fruits by-products is considered a valid approach to minimizing fruit waste as well as valorizing it through the production of food preservatives, flavors, and cosmetics^3^. Citrus waste contains valuable compounds in their pulp, seeds, and peels^4^, such as flavonoids, dietary fibers, polyphenols, carotenoids, ascorbic acids, and essential oil^2^.

Polyphenols and carotenoids are known to have various health benefits, especially their antioxidant activities, owing to their polyphenols contents presenting a variety of reported biological properties such as skin anti-agent, anti-carcinogenic, and anti-allergenicity^5^. In addition, they play a major role in the cosmetic and pharmaceutical fields. Essential oils (EOs) are a combination of many compounds but mainly consist of phenyl-propanoids, monoterpenes, and sesquiterpenes, which are responsible for the aroma of different plants, and can be used in pharmaceutical industries. They can be added to nutraceuticals to enhance flavor and can be used as natural antimicrobials^4^. Researchers found that citrus essential oils can control the growth of a broad range of bacteria without causing any detrimental health effects^6^.

Isolation of essential oils can be done conventionally using different methods such as; solvent extraction, cold pressing, and distillation^7^. Each conventional isolation method has some advantages and many disadvantages. Solvent extraction has a high yield but the solvents are expensive and difficult to remove^8^. Moreover, cold pressing has high-quality oil but of is low yield^9^. Nevertheless, conventional distillation methods have higher yield but are of low quality, and has a high energy consumption^10^. Moreover, there are many studies on the improvement of conventional hydrodistillation (HD), such as microwave-assisted extraction (MAE), pulsed-electric-field assisted HD (PEF-HD), and ohmic-assisted hydrodistillation (OAHD). However, the need for a special-apparatus, lower selectivity, and inevitable reaction in higher temperatures are the main MAE disadvantages^11^. Also, PEF-HD had its drawbacks which comprise the reversibility of the membrane-changes, air-bubbles making, which reduces the overall efficiency of the method^12^. Nevertheless, OAHD had several disadvantages including huge equipment cost, the risk of thermal-runaway, and inadequacy to direct heating of oil reservoirs^13^. Thus, there is an increasing need for a novel isolation method that shortens the isolation time, consumes lower energy, and is without carbon-dioxide production, with high yield and quality, and efficiency. Thus, the current work aims to phytochemically optimize a dual-function GC/MS-aided ultrasound-assisted hydrodistillation prototype (DF-GC/MS-HUS) utilizing *Citrus sinensis* essential oil (CS-EO) from byproducts. Moreover, the in-vivo antioxidant and in-vitro antibacterial potentials were also investigated.

## Materials and methods

### Plant materials

The *Citrus sinensis* (CS) zest, resulting from CS pressing, were collected (spring, 2019) and prepared from two different juice-joints located in Beirut, Tarik El Jdideh area, Lebanon. CS was freshly transported in well-sealed containers via private car in the shade. Zests from CS peels were obtained using a peeler (Pedrini, Model 6018-8AA, Italy). Experimental research and field studies on cultivated CS plants in Akar, Lebanon including the collection of plant material comply with relevant national cultivation and collection laws.

### Chemicals

Chemicals and solvents utilized in the conventional method study were of analytical grade. Moreover, chemicals, solvents, and standards used in the prototype method and GC analysis were of GC-grade. All chemicals, solvents, and standards were commercially purchased from Sigma-Aldrich (USA) unless stated. Double-distilled water was used in all experiments, whenever needed. Ethyl caprate, GC-internal standard, was purchased from Thermo Scientific Chemicals, USA.

### Dual-function GC/MS optimized ultrasound-assisted hydrodistillation prototype (DF-GC/MS-HUS) phytochemical method

**Figure 1:**
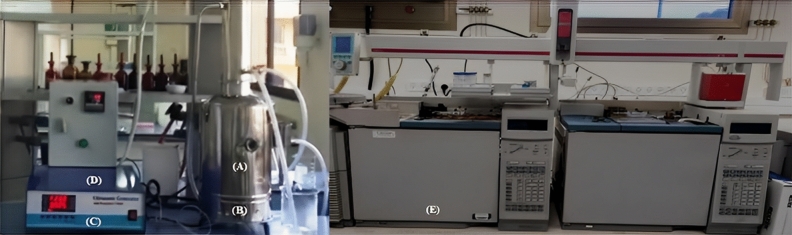
Dual-function GC/MS optimized ultrasound-assisted hydrodistillation prototype (DF-GC/MS-HUS). (**A**) Modified Clevenger hydro-distillator (upper-part). (**B**) Imbedded ultrasound transducer (lower-part). (**C**) Ultrasound control. (**D**) Central Control. (**E**) GC–FID–MS.

A Waters GC–FID–MS (Waters APNT1545129, Japan) was operated for performing the analysis in-line with the extraction process. The DF-GC/MS-HUS has been assembled of stainless-steel modified Clevenger hydro-distillator embedded with optimized four ultrasound transducers operated with central control for varying the ultrasound frequency, sonication, and distillation time, for maximum yield at the minimum time, according to the schematic representation in Fig. 1 and S1. The in-line split injector was used for separation and quantification of essential oils compounds^14^. The Waters GC–FID–MS was operated utilizing 5-MS capillary column (30 m * 0.25 mm * 0.25 μm) in addition to a Waters flame ionization detector (FID), and a mass detector operated in EI mode. The Hydrogen (FID), and Helium (MS), the carrier gases, flow managed at a rate of 1 mL/min, split ratio, 1:30, and adjusted at 250 °C. The temperature of the oven was programmed as follows; 50 °C at 5 °C per minute (5 min) 140 °C at 7 °C per minute and to 275 °C (10 min)^14^. The optimized Ultrasound-assisted hydrodistillation (HUS) had a dual-function in the extraction of the CS-EO. Primarily, HUS ultrasonic hydro-distillator was utilized as a source of adequate energy to break-open the oil-containing glands to release the CS-EO. Secondarily, HUS ultrasonic transducer was used as a dispersing-agent to emulsify the organic-phase in double-distilled water in the distillation flask. Three embedded ultrasound probes have been utilized with a 90–250 VAC power, and the pulsation cycle is set for 9.9 s. on and 9.9 s. off, and ca. avg. 0.2 VAC/sq cm power-density. We ensured no false positive by running a secondary TLC-UV confirmatory drug test on any major positive result^15^.

Mass spectral data were obtained using full scan mode (m/z 50–300), and a splitting ratio of 1:30. CS EO samples were diluted with n-hexane at a ratio of 1:500 (v/v), and ethyl caprate (internal standard, 0.38 mg/mL) were used for the analysis^16^.

Each sample was run in the following sequence: blank, CS EO sample, blank, CS EO sample, blank, CS EO, and blank at the end. Each sample was analyzed in triplicates and each run lasted 30 min. The identities of the separated-compounds have been assessed by comparing their retention times, retention indices, and mass spectra with those of available reference standards.

The other separated constituents obtained from the runs have been screened from the NIST mass spectral library based on their mass data and their identities were confirmed utilizing their linear-retention indices (LRI) relative to (C8–C20) n-alkanes.

### Conventional Clevenger hydrodistillation (CCH) method

The CS zest was immersed in 200 mL of distilled water in a ratio of 1:2, respectively. The zest was disposed of round-heater attached to a conventional Clevenger hydro-distillator (CCH) to ensure the isolation of CS-EO. The CCH was carried on for 6 h at 100 °C. And at the end of distillation, two phases were obtained; a water phase (aromatic water) and the E.O, less dense than water. The CS essential oil was stored at − 80 °C before analysis^17^.

#### Yield and isolation time

After peeling the CS to obtain the zest, the weight of the zest was taken using an electrical balance (Ohaus PR124ZH, China). The volume of CS oil isolated was taken using a micropipette after separating the water and oil phases through centrifugation (40 °C, under 400 rpm stirring). The yield of isolated CS E.O. was expressed in mL, and the isolation time by the two methods was expressed per hour^18^.

#### Kinetic modeling for the process of isolation

Currently, kinetic modeling for CS peel oil isolation by the prototype method was performed using the first-order and second-order models.

#### First order kinetic-model

The pseudo first-order kinetic equation: dCt/dt = k_1_(Cs − Ct), where Cs is the isolation-capacity, Ct is the concentration of essential oil at any time t (min), and k_1_ (min^−1^) is the first-order isolation rate constant. The Cs and k_1_ could be calculated utilizing the plot intercept and slope^19^.

#### Second order kinetic-model

The second-order kinetic equation: dCt/dt = k_2_(Cs − Ct)^2^, where k_2_ (L g^−1^ min^−1^) is the second-order isolation rate constant.

The initial isolation-rate (h), the isolation-capacity (Cs), and the second order isolation-rate constant (k_2_) can be estimated practically from the intercept and slope of a plot involving t/Ct and t^19,20^.

#### Biological, analytical, and biochemical analysis

Two commercial CS essential oils isolated using the conventional and DF-GC/MS-HUS methods were assessed separately biologically, analytically, and biochemically.

#### In-vivo and in-vitro antioxidant

The antioxidant potential was assessed utilizing in-vivo serum catalase (CAT), in-vitro Free radicals/1,1-Diphenyl-2-Picrylhydrazyl (DPPH), and in-vitro ABTS assay^21–23^. The in-vivo CAT levels have been measured (kU/l) by an adapted method described previously^23^. The in-vitro DPPH method was assessed utilizing different concentrations of all essential oils (25, 50, 100 μg/mL) which were diluted five times with DPPH solution in 0.4 mM methanol. The blank consisted of a 0.4 mM methanolic solution of DPPH. After 30 min of incubation at room temperature, the reduction in the number of free radicals will be measured by reading the absorbance at 517 nm using a spectrophotometer. The percentage inhibition of DPPH radicals by each E.O will be calculated according to the following formula: % of inhibition = [(AB − AA)/AB] × 100, Where AB absorption of the blank sample (t = 0) and AA = absorption of tested oil (t = 30 min). The half maximal inhibitory concentration: IC50 values which represents the concentration of E.O that caused 50% scavenging, will be determined from the plot of inhibition percentage against concentration^22^. The in-vitro ABTS assay levels have been measured by a method previously described^21^.

#### The refractive index (RI) analysis

The RI of both CS oil samples was measured using a REF 123® digital refractometer. Measurement was done at 20 ± 0.2 °C^14^.

#### The relative density analysis

According to^24^, the relative density of essential oil was calculated using the following formula the ratio of the mass of the liquid sample and the mass of water.

$$C=\frac{\mathrm{m}}{\mathrm{V}}$$

where C = density g/mL, m = mass per g, and V = volume per mL.

#### Total phenolic compounds analysis

According to the method described by Taga et al. (1984), 100 uL of each CS essential oil sample will be dissolved in 10 mL of 0.4 Mm MeOH, and 2 mL of this solution will be made up with 0.3% Hydrochloric acid to 5 mL. A 100 μL aliquot of the resulting solution will be added to 2 mL of 2% Sodium Carbonate (Na_2_CO_3_) and after 2 min, 100 μL of Folin–Ciocalteau reagent (diluted with MeOH 1:1) will be added and mixed well. After 30 min of incubation, the absorbance of the mixtures will be recorded spectrophotometrically at 750 nm according to the method used by^22^.

The total phenolic contents will be calculated as gallic acid equivalent (GAE) from a calibration curve of gallic acid standard solutions and expressed as mg of gallic acid per 100 μL of essential oil sample^22^.

### Microbiological analysis

#### Total counts

On a plate count agar (PCA) 50 μL of oil was added and rotated gently to ensure uniform mixing of the oil with agar. The plates are incubated at 30 °C for 48 h. The microbiological analysis of the oil was performed at the Microbiology laboratory, (Lebanese Agricultural Research Institute (LARI), Fanar, LEBANON).

#### Coliforms

On a violet-red bile agar, 50 uL of oil was added and spread on the agar. The plates are incubated at 37 °C for 24 h.

#### Antimicrobial effect

Two commercial CS essential oils and the extracted oil by the prototype were used in this study. The oils were first dissolved in dimethyl-sulfoxide (DMSO) 1:1 (v/v) to give stock solutions (50% v/v). For the bioassay, the stock solutions of essential oils were sterilized using 0.45 μm disposable syringe filters prior to the assessment of their antimicrobial effect. Stock solutions of essential oils were stored in dark bottles in the refrigerator at 4 °C for subsequent use.

#### Bacterial strains

The bacterial strains used in this study were provided by the Department of Health Sciences at Beirut Arab University (BAU) and the American University of Beirut Medical Center (AUBMC). Two gram-positive bacterial strains (*Staphylococcus aureus* and *Listeria monocytogenes*) and two gram-negative bacterial strains (*E.coli* and *Pseudomonas aeruginosa*) were tested against the two commercial CS essential oils and the extracted oil by prototype and were used to form mono-species bio-films. The cultures of bacteria were maintained in their appropriate agar slants at 4 °C throughout the study and used as stock bacterial cultures.

#### Inoculum standardization

Bacterial inoculums were obtained from stock cultures and inoculated in Lysogeny broth medium then incubated at 37 °C for 18–24 h. From the freshly grown cultures, decimal dilutions were made in sterile saline (0.9%) until reaching turbidity of 0.5 McFarland (10^8^ CFU/mL), for testing the antibacterial effects of essential oils^6^.

#### Agar disc diffusion method

The antibacterial activity of essential oils was first examined by the agar-disc diffusion method, which is the preliminary assay for screening the antibacterial activity of essential oils and selecting between efficient ones^25^. Agar disc diffusion was performed using an overnight bacterial culture, where 3 colonies of each strain to be tested were picked and inoculated in sterile saline solution and adjusted to approximately 10^8^ CFU/mL, then 100 µL of the prepared bacterial suspensions were spread over plates of Mueller–Hinton agar (MHA) and left for 15 min at room temperature. To enhance the diffusion in the agar, essential oils were mixed with 10% aqueous DMSO and sterilized by filtration through a 0.45 μm membrane filter^26^. Under aseptic conditions, 5 μL of essential oils in DMSO (1:1) were pipetted on the 5 mm sterilized filter paper discs placed on the top of inoculated MHA plates and kept for 30 min in the refrigerator to allow oil diffusion. Filter paper disc impregnated with DMSO was used as a negative control, while a standard disc containing gentamycin (10 μg/disc) was used as a positive control. All plates were incubated at 37 °C for 18–24 h. After overnight incubation, the inhibition zones were recorded^6^.

### Determination of minimum inhibitory concentration (MIC)

The MIC values were determined for the essential oils showing potent antibacterial effects against tested isolates using the agar dilution method as recommended by the National Committee for Clinical Laboratory Standards (NCCLS) with some modifications^6^. To enhance the solubility of essential oils, a final concentration of 0.5% (v/v) Tween-20 was added to the MHA medium. First, stock solutions of each tested essential oil (100 mg/mL) were prepared, followed by a series of two-fold dilutions in Mueller–Hinton broth resulting in six concentrations (50 mg/mL, 25 mg/mL, 12.5 mg/mL, 6.25 mg/mL, 3.12 mg/mL and 1.56 mg/mL), then 1 mL of each prepared serial dilution was added to 9 mL of melted Mueller Hinton agar at 48 °C, mixed thoroughly and poured in sterile plates. Plates were dried at room temperature for 30 min prior to the spot with 3 μL aliquots of bacterial cultures containing approximately 10^4^ CFU/mL of each tested isolate. Inoculated plates were incubated at 37 °C for 18–24 h and the MICs values were determined. Plates of MHA without essential oils were used as negative growth control. The MICs values were considered as the lowest concentration of oil resulting in the inhibition of visible bacterial growth on the agar plate^27^.

### Mono-species biofilm formation

To assess the effect of essential oils on biofilm formation, biofilm on glass surface assay was performed, where the adhered biofilm to glass coverslips is visualized under the light microscope^28^. Each bacterial strain was grown overnight in nutrient broth and diluted 1:5 in Luria–Bertani broth (LB), diluted cultures were used to immerse a sterile coverslip. In this study, sterile beakers for each bacterial strain containing a 2.5 cm coverslip were used, 300 μL bacterial suspension was added with an equal volume of essential oil showing a potent antibacterial effect against each isolate, and the untreated beaker was used as a reference control. After overnight incubation at 37 °C, each beaker was washed 3 times with distilled water, fixed with 95% of ethanol for 30 min, and then stained with 0.1% crystal violet for one hour at room temperature. After a final wash, all coverslips were dried and microscopically visualized for bio-film formation, and all tests were conducted in triplicate^6^.

### Statistical analysis

The results are expressed as mean ± SD. Analysis of variance (ANOVA) has been utilized to test for differences in the groups and multiple-range test of significance has been used. The Tukeys’ test was applied to examine the means at a *p* < 0.05. The OriginPro 2021 program (Origin Lab; Northampton, MA, USA) was used for graphical presentations.

## Results and discussion

An enormous amount of citrus by-products is being wasted every-year. There is an increasing need to utilize these by-products with high-efficiency. Thus, this study focuses on the EO-isolation from *Citrus sinensis* (CS) by-products, using a novel dual-function GC/MS optimized ultrasound-assisted hydrodistillation-prototype. In this study, the isolation of essential oil from CS by-product, using a novel dual-function GC/MS optimized ultrasound-assisted hydrodistillation prototype (DF-GC/MS-HUS). The CS essential oil (CS-EO) was GC analyzed by MS-detector (GC/MS) and optimized by flame-ionization detector (GC/FID).

### Dual-function GC/MS optimized ultrasound-assisted hydrodistillation prototype (DF-GC/MS-HUS) phytochemical analysis

To maximize the yield at the minimum time, the DF-GC/MS-HUS prototype was optimized by varying the ultrasound frequency, sonication, and distillation time. The details of the main DF-GC/MS-HUS optimized conditions were summarized in Table S1. The most effective DF-GC/MS-HUS optimized conditions were isolation under 38 °C of optimized-temperature and 10 min of 28.9 Hz sonication, and 60 min of distillation. The yield was 8.80 ± 0.01 mL CS-EO (prototype) per hour (Table 1). The relatively higher-yield might be due to Ultrasound-assisted hydrodistillation (HUS) having a dual-function in the extraction of the CS-EO. Primarily, HUS was utilized as a source of adequate energy to break-open the oil-containing glands to release the CS-EO. Secondarily, HUS was used as a dispersing-agent to emulsify the organic-phase in double-distilled water. To assess the efficiency of the prototype method, the prototype CS EO was analyzed by GC–MS for separation and identification of CS EO components. The in-line GC/MS indicated that the main components of the isolated CS-EO (prototype) were limonene (81.32%), β-myrcene (7.55%), and α-pinene (4.20%) (Table 4).

**Table 1:** Comparison between the isolation time and the yield of various methods.

	Conventional CCH	DF-GC/MS-HUS Prototype**
Time	6.00 ± 0.01 h	1.00 ± 0.01 h*
Yield	3.5 × 10^–4^ ± 0.01 mL	8.80 ± 0.01 mL*

*indicates a significant difference between Conventional CCH and prototype methods (*p* < 0.05).

**Dual-function GC/MS optimized ultrasound-assisted hydrodistillation prototype.

### Conventional Clevenger hydrodistillation (CCH) method

The CS-EO was isolated from CCH (CS-EO Conventional), and the yield was 0.35 μL per 6 h of distillation. The CS-EO (Conventional) GC/MS analysis has shown a decline in main components quantity and an increase of artifacts, indicating an overall lower quality of the oil. This inferiority in CS-EO quality might be due to the utilization of high-temperature, and longer time of CS-EO extraction. To estimate the efficiency of the CCH method, the CCH CS EO was analyzed by GC–MS to evaluate the CCH CS EO components. The main components of the conventional method have shown limonene (60.23%), β-myrcene (5.33%), and α-pinene (2.10%) (Table 4).

### DF-GC/MS-HUS prototype versus conventional CCH method

A difference in the extraction time was noted between the DF-GC/MS-HUS prototype and the conventional CCH (Table 1). The conventional CCH hydrodistillation required 6 h to extract a volume of 0.35 μL, while the DF-GC/MS-HUS prototype isolation optimized for 60 min to give a 2514-folds increase in yield. Thus, the DF-GC/MS-HUS prototype has shown superiority in extraction efficiency. When compared to the conventional hydrodistillation method, the DF-GC/MS-HUS prototype method has shortened the extraction time by 83.3%, lowered energy consumption, without carbon-dioxide production, by reducing extraction temperatures by more than half, which protected the thermolabile components, and increased the quantity by 2514 folds, and improved the quality of CE-EO composition. To evaluate the efficiency of the prototype method with conventional-hydrodistillation, the essential oils by these two methods were analyzed by GC–MS for separation and identification of CS EO components. The main components of the isolated prototype CS-EO were limonene (81.32%), β-myrcene (7.55%), and α-pinene (4.20%). On the other hand, the conventional method has shown limonene (81.32%), β-myrcene (5.33%), and α-pinene (2.10%) as the conventional main components. Therefore, the DF-GC/MS-HUS prototype method is considered a novel green-technique that minimized the utilization of energy with higher efficiency. These results were comparable to other optimized systems reported previously^4^.

**Figure 2:**
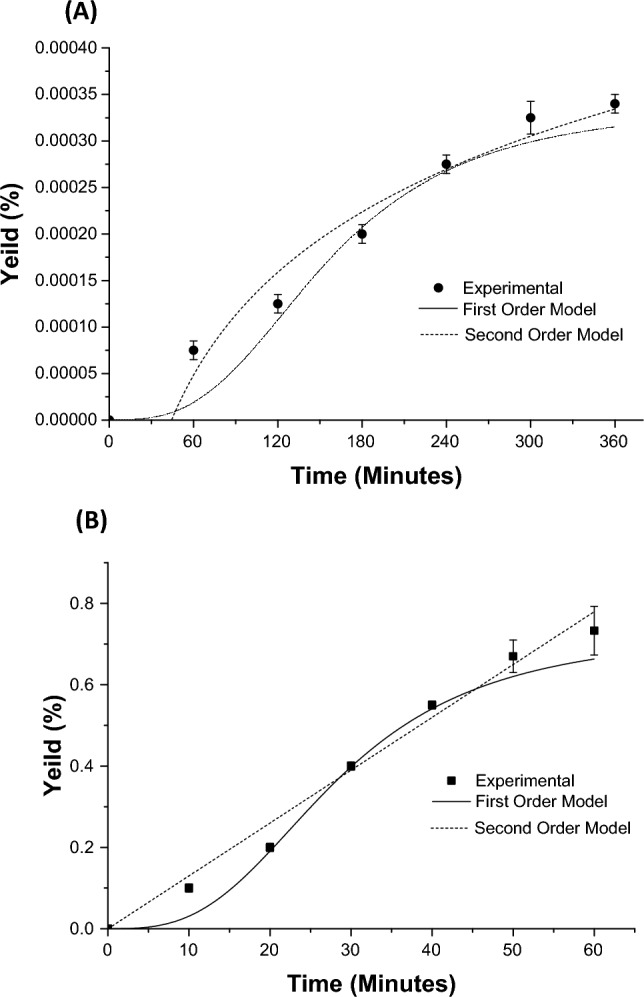
A comparison of the first-order and second-order kinetic models with the experimental results for the extraction of orange oil by (**A**) Conventional hydrodistillation method and (**B**) Dual function GC/MS optimized Ultrasound-Assisted Hydrodistillation Prototype method (DF-GC/MS-HUS).

The first-order and the second-order kinetics have been studied utilizing the CCH and the DF-GC/MS-HUS prototype methods (Fig. 2). When kinetic modeling was performed, it was found that the second-order kinetic model was capable to represent the practical results of CS oil extraction using the conventional hydrodistillation and the prototype methods when compared with the first order kinetic model (Tables 2, 3). Moreover, yield % could be used to show that second-order kinetic model was capable to represent the practical results of CS oil isolation using the conventional hydrodistillation and the prototype methods when compared with the first order kinetic model, as previously accounted with similar essential oils^19^. Currently, the electric consumption requirements for CS oil extraction using the conventional method (hydrodistillation) and the prototype method were 7.10 and 1.18 kW h, respectively. Thus, it could be concluded that the CS oil extraction using the prototype method conserved the energy by 6 folds when compared to the conventional hydrodistillation method^29^. These results are in-line with other optimized systems reported previously with similar essential oils^4,30^.

**Table 2:** Linearization of the first order kinetic model for CS essential oil isolation using conventional CCH, and Dual-function GC/MS optimized ultrasound-assisted hydrodistillation prototype (DF-GC/MS-HUS) methods.

Isolation methods^a^	Slope	k_1_ (min^−1^)	Intercept	Cs, (g L^−1^)	R^2^
Conventional CCH	− 0.058 ± 0.010	0.009 ± 0.010	0.001 ± 0.001	0.056 ± 0.001	0.84872 ± 0.001
DF-GC/MS-HUS Prototype	− 0.016 ± 0.010*	0.052 ± 0.010*	0.013 ± 0.001*	0.852 ± 0.001*	0.8691 ± 0.001

^a^Values obtained from OriginPro.

*Indicates a significant difference between Conventional CCH and prototype methods (*p* < 0.05).

**Table 3:** Linearization of the second order kinetic model for CS essential oil isolation using conventional CCH, and Dual-function GC/MS optimized ultrasound-assisted hydrodistillation prototype (DF-GC/MS-HUS) methods.

Isolation methods^a^	Slope	Cs (g L^−1^)	Intercept	K_2_ (L g min^−1^)	R^2^
Conventional CCH	9.94 × 10^−^ ± 0.001	0.020 ± 0.001	− 3.341 × 10^–7^ ± 0.001	0.0673 ± 0.0001	0.9940 ± 0.0001
DF-GC/MS-HUS Prototype	0.013 ± 0.001*	0.890 ± 0.001*	− 8.367 × 10^–8^ ± 0.001*	0.0235 ± 0.0001*	0.9785 ± 0.0001

^a^Values obtained from OriginPro.

* indicates a significant difference between Conventional CCH and prototype methods (*p* < 0.05).

Generally, the environmental impact of the E.Os extraction could be seen from the produced carbon dioxide emissions. The carbon dioxide emissions produced in CS oil extraction using the conventional CCH method, and the DF-GC/MS-HUS prototype method are equal to 4.9 and 0.96 kg, respectively. Thus in general it can be said that the CS oil extraction using the conventional CCH method (hydrodistillation) produces five folds much higher carbon dioxide emissions when compared with the prototype method. Thus, the use of the DF-GC/MS-HUS prototype method for CS oil extraction could be considered to be a new green technique compared to conventional CCH methods^29^.

While comparing the quality of the isolated essential CS oil using CCH and the prototype methods of isolation, the main components of the isolated CS-EO were limonene (81.32%), β-myrcene (7.55%), and α-pinene (4.20%) were predominate with the DF-GC/MS-HUS prototype method. This might be due to the advantage of the prototype since the oil is isolated for a shorter time at a lower temperature when compared to the conventional CCH method. With reference to the environmental-impact, the calculated quantity of carbon dioxide rejected in the atmosphere is higher in the case of conventional method (ca. 3464 g CO2/g of EO) than for prototype (ca. 199 g CO2/g of EO) (Table S2), as previously establish before^31^. According to these findings, the prototype method has improved essential CS oil quality while utilizing less energy, lower time consumption, lower carbon dioxide emission, and higher yield. Thus, the DF-GC/MS-HUS prototype method is considered a novel green-technique that minimized the utilization of energy with higher efficiency, as evidenced before with comparable techniques^32–34^.

### Biological, analytical, and biochemical analysis

CS essential oils isolated using the conventional and DF-GC/MS-HUS methods were assessed separately biologically, analytically, and biochemically (Table 4).

**Table 4:** The chemical composition of oils extracted using the dual-function GC/MS optimized ultrasound-assisted hydrodistillation prototype (DF-GC/MS-HUD prototype), and the conventional Clevenger hydrodistillation (CCH) methods.

Composition (%)				
Chemical nature/no	RT	Compounds	CCH EO	DF-GC/MS-HUD EO
Alcohols				
1	5.73	1-Octanol	0.08 ± 0.01	0.15 ± 0.01*
2	6.04	Linalool	1.28 ± 0.01	2.24 ± 0.01*
3	7.27	*Trans-p*-Mentha-2,8-dien-1-ol	0.04 ± 0.01	1.20 ± 0.01*
4	7.44	*Cis-p*-Mentha-2,8-dien-1-ol	Tr	2.40 ± 0.01*
5	8.00	α-Terpineol	0.40 ± 0.01	1.10 ± 0.01*
6	8.23	*(E)*-Carveol	0.03 ± 0.01	2.20 ± 0.01*
7	8.29	*(Z)*-Carveol	0.06 ± 0.01	1.11 ± 0.01
8	8.96	*p-*Mentha-1,8-dien-9-ol	0.05 ± 0.01	0.33 ± 0.01
Aldehydes				
9	7.08	Nonanal	0.14 ± 0.01	0.15 ± 0.01
10	5.05	Octanal	0.57 ± 0.01	Tr
11	7.54	Citronellal	0.20 ± 0.01	Tr
12	8.09	Decanal	0.55 ± 0.01	0.44 ± 0.01
13	8.42	β-Citral	0.20 ± 0.01	0.18 ± 0.01
14	8.71	α-Citral	0.25 ± 0.01	0.20 ± 0.01
15	9.12	Dodecanal	0.15 ± 0.01	Tr
16	12.96	β-Sinensal	0.13 ± 0.01	Tr
17	14.54	α-Sinensal	0.08 ± 0.01	Tr
18	8.81	Perillaldehyde	0.08 ± 0.01	0.40 ± 0.01
Ketone				
19	8.49	Carvone	0.05 ± 0.01	3.40 ± 0.01*
Terpenes				
20	4.34	α-Pinene	2.10 ± 0.01	4.20 ± 0.01*
21	4.75	Sabinene	1.13 ± 0.01	2.24 ± 0.01*
22	4.90	β-Myrcene	5.33 ± 0.01	7.55 ± 0.01*
23	5.13	3-Carene	0.54 ± 0.01	0.74 ± 0.01*
24	4.49	Limonene	60.23 ± 0.01	81.32 ± 0.01*
25	9.79	α-Copaene	0.11 ± 0.01	Tr
26	9.90	β-Cupepene	0.12 ± 0.01	Tr
27	9.95	Valencene	0.20 ± 0.01	0.38 ± 0.01
28	11.05	α-Farnesene	0.05 ± 0.01	0.99 ± 0.01*
Epoxide				
29	7.37	Limonene 1,2-epoxide	Tr	2.88 ± 0.01*
Total			96.03 ± 0.01	97.17 ± 0.01

*Indicates a significant difference between Conventional CCH and prototype methods (*p* < 0.05).

“tr.” Indicates less than 0.01, RT, retention times.

### In-vivo and in-vitro antioxidant

**Figure 3:**
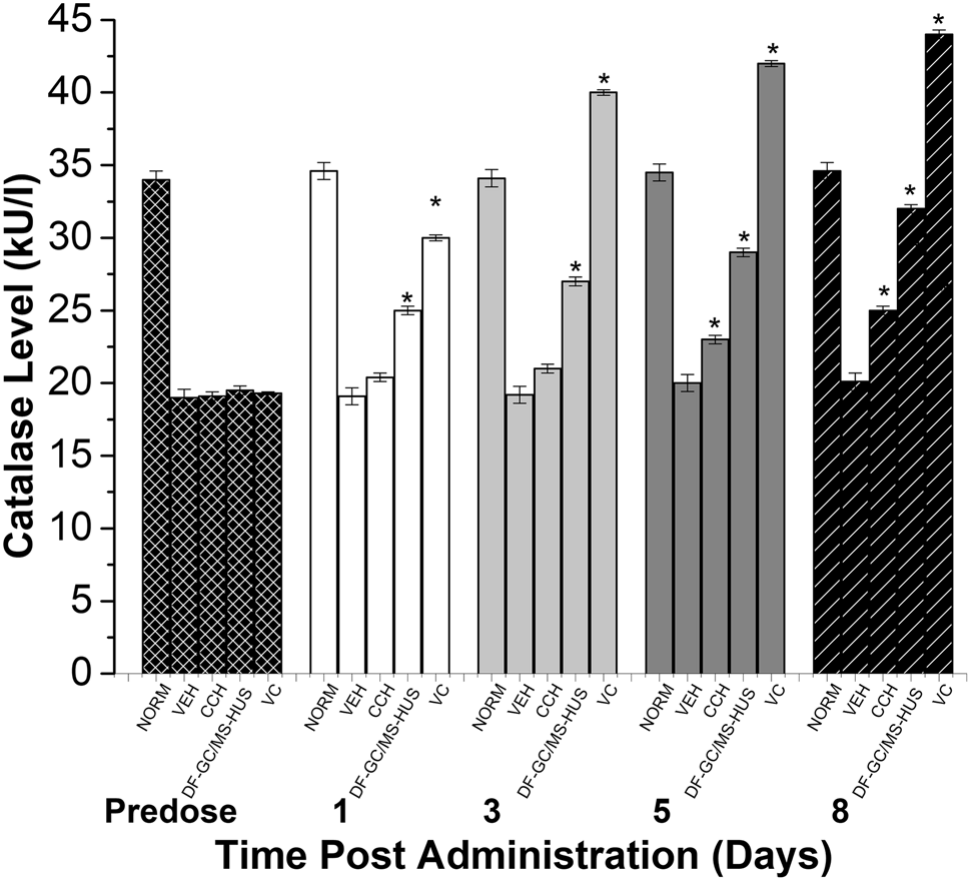
In-vivo CAT antioxidant analysis. *NORM* normal animals, *VEH* vehicle treated animals, *CCH* conventional Clevenger hydro-distillation (10 mg/kg), *DF-GC/MS-HUS* dual function GC/MS optimized ultrasound-assisted hydrodistillation prototype (10 mg/kg), *VC* 7 mg/kg vitamin C.

The in-vivo CAT levels are responsible for normal cell reduction of oxidative damage which initiates neuropathic pain. CAT serum levels have been evaluated before and eight-weeks after administration of 10 mg/kg of either CS oil isolated via conventional CCH and DF-GC/MS-HUS methods (Fig. 3). After eight weeks of CS oil (DF-GC/MS-HUS) 10 mg/kg administration, the CAT-levels have shown a 59.57 ± 0.55% elevation (Fig. 3). On the other hand, the CAT-levels have shown a 24.10 ± 0.14% elevation, after eight weeks of conventional CS oil (Fig. 3).

**Figure 4:**
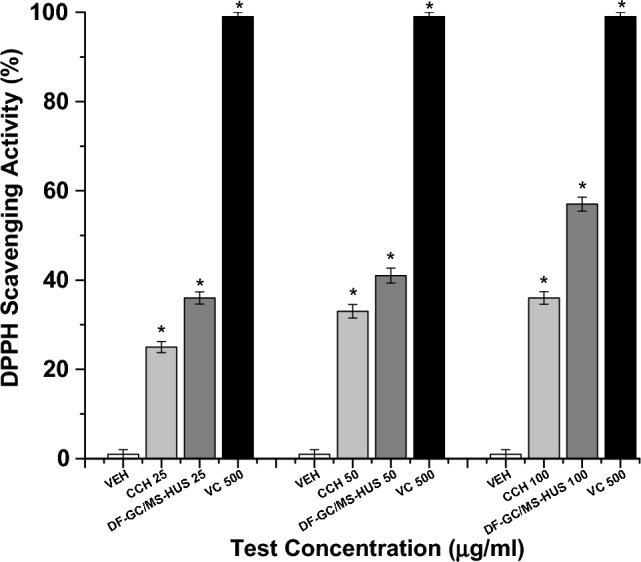
DPPH scavenging assay. *VEH* vehicle control, *CCH* conventional Clevenger hydro-distillation isolated essential oil, *DF-GC/MS-HUS* dual function GC/MS optimized Ultrasound-Assisted Hydrodistillation Prototype isolated essential oil, *VC* 500 μg/mL vitamin C, “*” means significant (*p* < 0.05) when compared to vehicle control (n = 3).

According to the DPPH free radical scavenging rate of the CS E.O in different concentrations, the oil obtained by the DF-GC/MS-HUS prototype and conventional CCH isolated essential oils, and as shown in (Fig. 4), the DPPH scavenging activity of oil increased significantly (*p* < 0.05) when increasing the concentration of oil. The DF-GC/MS-HUS prototype isolated CS E.O presents the highest percentage of free radical scavenging rate in all concentrations 36.00 ± **0.21**%, 41.00 ± **0.15**%, and 57.00 ± **0.26**% in 25 μg/mL, 50 μg/mL, and 100 μg/mL, respectively (Fig. 4). The conventional CCH isolated EO has shown to have the lowest % of DPPH radical scavenging activity, 10.00 ± **0.05**%, 11.50 ± **0.11**%, and 14.80 ± **0.05**% in 25 ug/mL, 50 ug/mL and 100 μg/mL, respectively (Fig. 4). The DF-GC/MS-HUS prototype CS E.O. has an IC_50_ of 87.7 μg/mL, the conventional CCH isolated EO has an IC_50_ of 337.83 μg/mL. The prototype extracted CS E.O showed the highest anti-radical activity with a limonene percentage of 60.23%. Thus, the prototype oil had a lower IC_50_ value with a higher antioxidant activity of 57% at 100 μL concentration, as previously observed with similar natural compounds^35^. The in-vitro ABTS assay results confirmed the DPPH results (Fig. S2).

The refractive index is calculated to determine the purity of the E.Os, the values of the conventional CCH and DF-GC/MS-HUS isolated essential oils present a RI range between 1.4705 and 1.4725 as shown in Table 5, and according to the essential oil association (EOA), the RI standards of essential oils should fall between a range of 1.4723–1.4737. The RI of the extracted CS oil (1.4725) fall within the range specified by EOA, and agrees with other studies done before on other CS essential oils^29,36^. The relative density values of various CS E.Os samples are nearly the same, as shown in Table 5. Moreover, according to the International Organization of Standardization (ISO 4735–2002 standards), the relative density of essential oils should fall within a range between 0.848 and 0.855 g/mL (“ISO—ISO 4735:2002—Oils of Citrus—2002.). The relative density of various CS E.Os samples range between 0.849 and 0.852 falling in the ISO-specific range. Our findings are also in line with a study^37^, that found the range of refractive index between 0.845 and 0.851. Total phenolic equivalent was determined using the Folin-Ciocalteu reagent and expressed as gallic acid equivalent in mg/100 μL and the results are presented in Table 5. The total phenolic equivalent ranged from 0.291 to 0.783 mg/100 mL E.O. The total phenolic equivalent was more in the DF-GC/MS-HUS prototype isolated CS EO than the CCH isolated CS EO (Table 5), and several studies report that the higher the phenolic content the higher the antioxidant activity in essential oils^38^. Thus, the prototype isolated oil presented the highest polyphenol contents of 0.783 mg/100 mL (Table 5), and showed a better antioxidant activity of 57.0 ± 0.01% utilizing the in-vitro DPPH method, and 58.90 ± 0.01% using the in-vivo CAT method (Figs. 3, 4). On the other hand, the conventional CCH EO has shown less polyphenol contents 0.362 mg/100 mL (Table 5), and showed a comparatively less antioxidant activity of 36.00 ± 0.01% utilizing the in-vitro DPPH method, and 23.60 ± 0.01% using the in-vivo CAT method (Figs. 3, 4). Therefore, the prototype oil was predominant in phenolic content, in-vitro, and in-vivo antioxidant potentials, when compared to the conventional isolation method.

**Table 5:** The Refractive Index, Relative Density, and total phenolic in the CS essential oil samples.

Sample	Refractive index	Relative density (g/mL)	Polyphenol content (mg/100 mL)
Conventional CCH Isolated EO	1.4720 ± 0.02	0.852 ± 0.01	0.362 ± 0.01
DF-GC/MS-HUS Prototype** Isolated EO	1.4725 ± 0.01	0.849 ± 0.01	0.789 ± 0.01*

*Indicates a significant difference between Conventional CCH and prototype methods (*p* < 0.05).

**Dual-function GC/MS optimized ultrasound-assisted hydrodistillation prototype.

**Figure 5:**
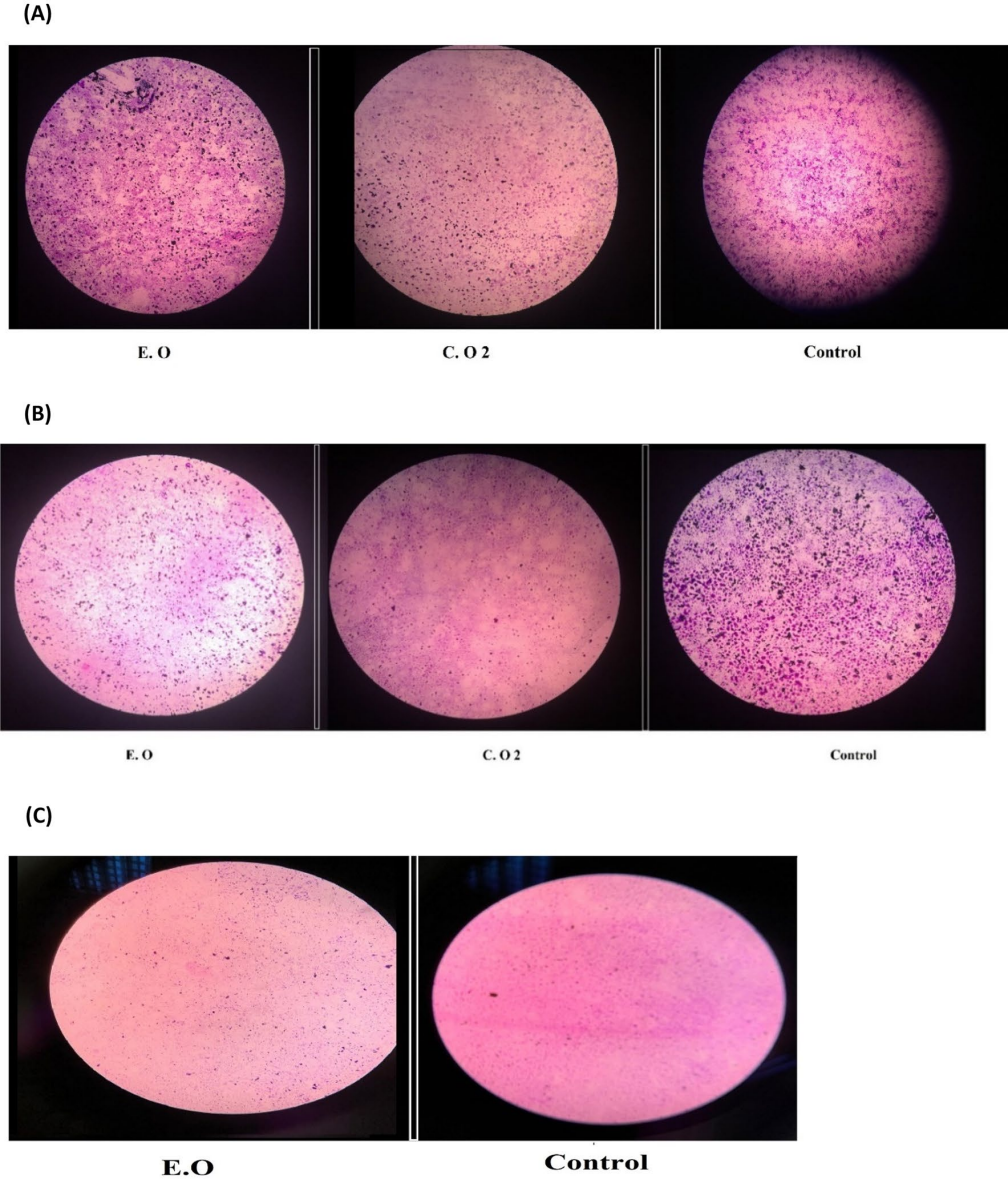
Microscopic visualization of the effect of E.Os on biofilm (**a**) *Staphylococcus aureus* (**b**) *Listeria monocytogenes* (**c**) *E.coli* compared to control. C. O2: conventional Clevenger hydro-distillation isolated essential oil. E.O: Dual function GC/MS optimized Ultrasound-Assisted Hydrodistillation Prototype isolated essential oil.

Various CS EO samples were free from bacteria, as various EO samples have shown no bacterial growth. Moreover, the DF-GC/MS-HUS and CCH isolated CS EOs antibacterial activities were assessed separately against four bacterial strains, studied by disc diffusion method (Table 6). The various CS EO inhibited bacterial growth significantly (*p* < 0.05) utilizing the disc diffusion method with variable effectiveness. The prototype oil was the only oil that showed activity against *E. coli* (11 mm growth inhibition zone). The conventional and the prototype oils have shown significant antibacterial activity on gram positive bacteria ranging from 8 to 13 mm, followed by the gram negative bacterial strains with a maximum antibacterial activity of 11 mm (Fig. 5). This is due to the difference in the structure of their cell wall, making gram negative bacteria more resistant to E.O than gram positive bacteria, these results were in agreement with previous studies^38^. The significant antibacterial activities of both the conventional and the prototype oils might be due to significant high limonene and oxygenated monoterpene contents^39^. Both oils have shown approximately the same effect on *L. monocytogenes* (8 mm and 9 mm respectively) and on *S. aureus* (13 mm and 11.5 mm respectively), with no antibacterial effect on *P. aeruginosa* (Fig. 5). Our results coincide with previously reported studies^32,40^. The most promising CS essential oils showing an antibacterial effect against tested gram positive and gram negative bacterial strains were selected for the determination of MIC values (presented in Table 6) since MIC values showed variability between the same types of E.Os^41^. In this study, the MIC of the prototype CS E.O for *E. coli* recorded a high value (50 mg/mL) when compared with the MIC value (2.5 mg/mL) for gram positive bacteria (*S. aureus* and *L. monocytogenes*), our results coincide with other studies showing that essential oils showed variability in their antimicrobial activity among different microorganisms^6^. Several factors may cause a difference in the antibacterial potential of essential oil, such as the variability of oil compounds and the structure of bacterial cell walls^42^. Moreover, the prototype EO has shown a greater inhibitory effect against gram positive bacteria than the conventional oil, with MIC values of 3.125 mg/mL for *S. aureus* and 12.5 mg/mL for *L. monocytogenes*. This could be related to the higher percentage of limonene in the prototype EO^41^.

**Table 6:** Average inhibition zone diameter (mm) of various CS isolated essential oils (EO) against different bacterial strains using disc diffusion method, and the Minimum inhibitory concentration (mg/mL) on selected bacterial strain.

Bacterial strains	Conventional CCH EO	DF-GC/MS-HUS^b^ Prototype EO	Gentamicin 10 μg
*Staphylococcus aureus*	11.5 ± 0.11	13.0 ± 0.12*	26 ± 0.20
MIC (mg/mL)	3.125 ± 0.01	2.50 ± 0.20*	–^a^
*Listeria monocytogenes*	8.0 ± 0.13	9.0 ± 0.12	26 ± 0.21
MIC (mg/mL)	12.5 ± 0.11	2.50 ± 0.23*	–
*E.coli*	–	11.0 ± 0.12*	21 ± 0.24
MIC (mg/mL)	NA	50.0 ± 0.29*	–
*Pseudomonas aeruginosa*	–	–	19.0 ± 0.12

*Indicates significant difference between Conventional CCH and prototype methods (*p* < 0.05).

^a^–: Absence of inhibition zone.

^b^Dual-function GC/MS optimized ultrasound-assisted hydrodistillation prototype.

In the nutraceuticals industry, the bacterial bio-film represented a severe hygiene problem, rendering gram positive and gram negative bacteria more resistant to disinfectants and antimicrobial agents^43^. Many E.O s have shown potent suppression of bio-film formation by promoting cell separation^44^. Our results have shown that both, the conventional and the prototype, EOs were able to interrupt the bio-film formation of the three tested bacterial strains (*E.coli*, *S. aureus*, and *L. monocytogenes*) resulting in a significant decrease in cell attachment on the surface of glass cover-slips (Fig. 5). Our results are consistent with other studies showing that bio-films were strongly inhibited by the E.Os where the sub-lethal damage of the cell wall can negatively influence the first step in bio-film formation which is the bacterial attachment to surfaces^43^. Thus, the prototype and the conventional isolated EOs have shown significant disruption of the bacterial bio-film, which has a practical application in the maintaining hygiene of the nutraceuticals industry in a safe natural way.

## Conclusion

In this study, CS essential oils were isolated utilizing the DF-GC/MS-HUS prototype and the CCH conventional methods. The two various methods were compared on the extraction time, yield, and essential oil composition. The higher yield of oil was obtained using the prototype technique, improving the oil composition, with minimal isolation time and energy consumption, leading to a reduced burden on the environment. In-vitro and in-vivo antioxidant potentials, total polyphenol, refractive index, relative density, antibacterial activity, and bio-films inhibition were also assessed. The main components of the isolated prototype CS-EO were limonene (81.32%), β-myrcene (7.55%), and α-pinene (4.20%). Both investigated oils produced significant in-vitro and in-vivo antioxidant potentials, with the highest one being for the prototype isolated CS EO. The prototype oil was predominant in phenolic content, in-vitro, and in-vivo antioxidant potential, when compared to the conventional isolation method. The prototype EO significant anti-oxidant potential might be due to its higher content of total polyphenol, when correlated to the conventional EO. The refractive indices and relative density values of the two oil samples fall in the specified range according to EOA and ISO standards, respectively. Both essential oils have shown to be bacteria free, and have natural antibacterial potentials to minimize bacterial growth and inhibit bio-film formation by *S. aureus*, *L. monocytogenes*, and *E.coli*. The prototype and the conventional isolated EOs have shown significant disruption of the bacterial bio-film, which has a practical application in maintaining hygiene of the nutraceuticals industry in a safe natural way. When compared to the conventional hydrodistillation method, the DF-GC/MS-HUS prototype method has shortened the isolation time by 83.3%, lowered energy consumption, without carbon-dioxide production, by reducing extraction temperatures by more than half, which protected the thermolabile components, and increased the quantity by 2514 folds, and improved the quality of CE-EO composition. Therefore, the DF-GC/MS-HUS prototype method is considered a novel green-technique that minimized the utilization of energy with higher efficiency. More experiments would be done in the future to evaluate other green methods for the valorization of natural byproducts.

## Supplementary Information

Supplementary Information.

## Data Availability

The datasets analyzed during the current study are not publicly available due to an agreement with LIRA funding agency but are available from the corresponding author on reasonable request.

## Abbreviations

EO: Essential oils
CS: *Citrus sinensis*
DF-GC/MS-HUS: Ultrasound-assisted hydrodistillation prototype
Prototype: Ultrasound-assisted hydrodistillation prototype
CCH: Conventional Clevenger Hydro-distillation
Μl: Microliter
IC5O: The half-maximal inhibitory concentration
MRL: Maximum residues limit
MIC: Minimum inhibitory concentration
RI: Refractive index
MHA: Mueller Hinton agar
CO2: Commercial CS essential oil number two
EOA: Essential Oil Association
ISO: International Organization of Standardization
Cs: The isolation-capacity
Ct: The concentration of essential oil at any time t (min)
k_1_: The first-order isolation rate constant
k_2_: The second order isolation-rate constant
