# Supplementary material for: Novel dual-function GC/MS aided ultrasound-assisted hydrodistillation for the valorization of Citrus sinensis by-products: phytochemical analysis and anti-bacterial activities
Source: Sci Rep. 2023 Aug 2;13:12547. doi: 10.1038/s41598-023-38130-9 (PMC10397203; doi:10.1038/s41598-023-38130-9)
Supplement: Supplementary file 1 — Supplementary Information. [file 41598_2023_38130_MOESM1_ESM.docx]

Novel Dual-Function GC/MS aided Ultrasound-Assisted Hydrodistillation for the Valorization of *Citrus sinensis* by-products: Phytochemical Analysis and Anti-bacterial Activities

**Supplementary File**

**
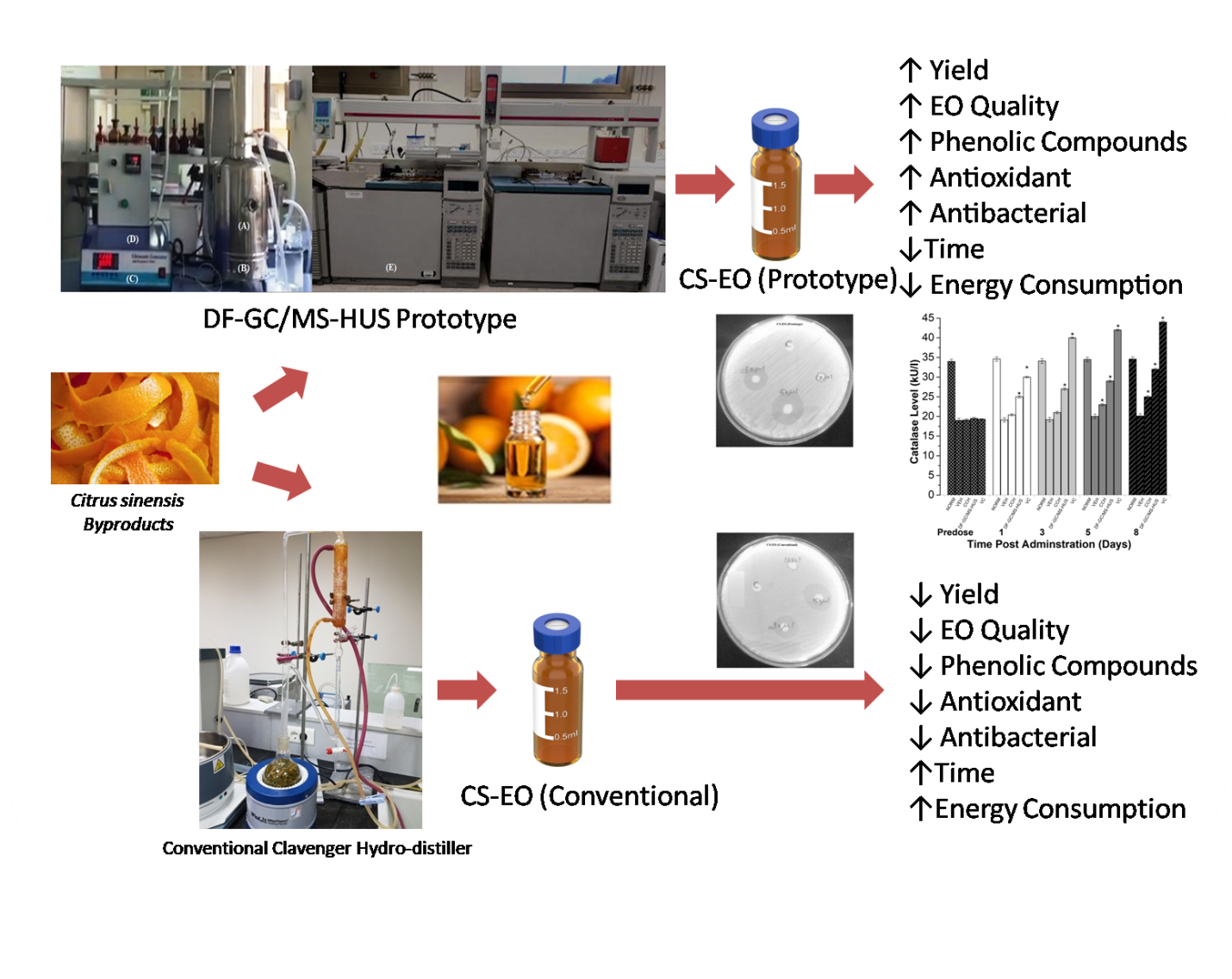
**

Figure S1. The graphical abstract illustrating the novel dual-function gc/ms aided ultrasound-assisted hydrodistillation for the valorization of citrus sinensis by-products.

Fig. S2. ABTS scavenging assay. VEH: vehicle control. CCH: conventional Clavenger hydro-distillation isolated essential oil. DF-GC/MS-HUS: Dual function GC/MS optimized Ultrasound-Assisted Hydrodistillation Prototype isolated essential oil. VC: 500 μg/ml vitamin C. “*” means significant (*p*<0.05) when compared to vehicle control (n=3).

Table S1. Details of the main effective DF-GC/MS-HUS optimized conditions.

| Optimizations | Temperature (°C) | Sonication (Hz) | Time (min) | Yield (mL) | GC-MS-FID |
| --- | --- | --- | --- | --- | --- |
| T1 | 30 | 14.45 | 30 | 3.40 ± 0.01 | limonene (24.10%), β-Myrcene (3.02%), and α-Pinene (1.70%) |
| T2 | 34 | 14.45 | 30 | 4.60 ± 0.01 | limonene (31.10%), β-Myrcene (3.55%), and α-Pinene (1.90%) |
| T3 | 38 | 14.45 | 30 | 4.90 ± 0.01 | limonene (33.13%), β-Myrcene (4.15%), and α-Pinene (2.30%) |
| T4 | 38 | 28.9 | 30 | 5.40 ± 0.01 | limonene (37.10%), β-Myrcene (4.60%), and α-Pinene (2.50%) |
| T5 | 38 | 28.9 | 60 | 8.80 ± 0.01 | limonene (60.23%), β-Myrcene (7.55%), and α-Pinene (4.20%) |

Table S2. Comparison between the carbon dioxide emissions of various methods.

|  | Conventional CCH | DF-GC/MS-HUS Prototype |
| --- | --- | --- |
| Carbon Dioxide Emission | 3464.0 ± 29.0 g CO2/g of EO | 199.0 ± 1.0 g CO2/g of EO* |

* indicates a significant difference between Conventional CCH and prototype methods (p <0.05)
